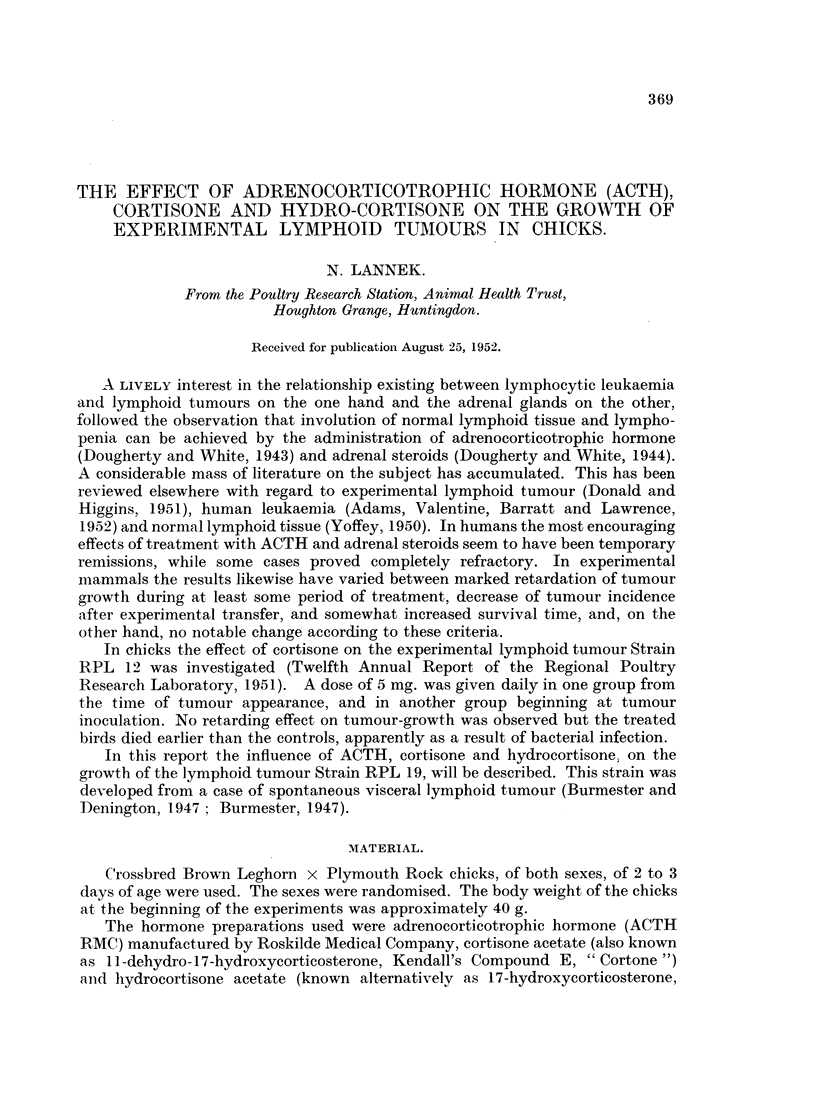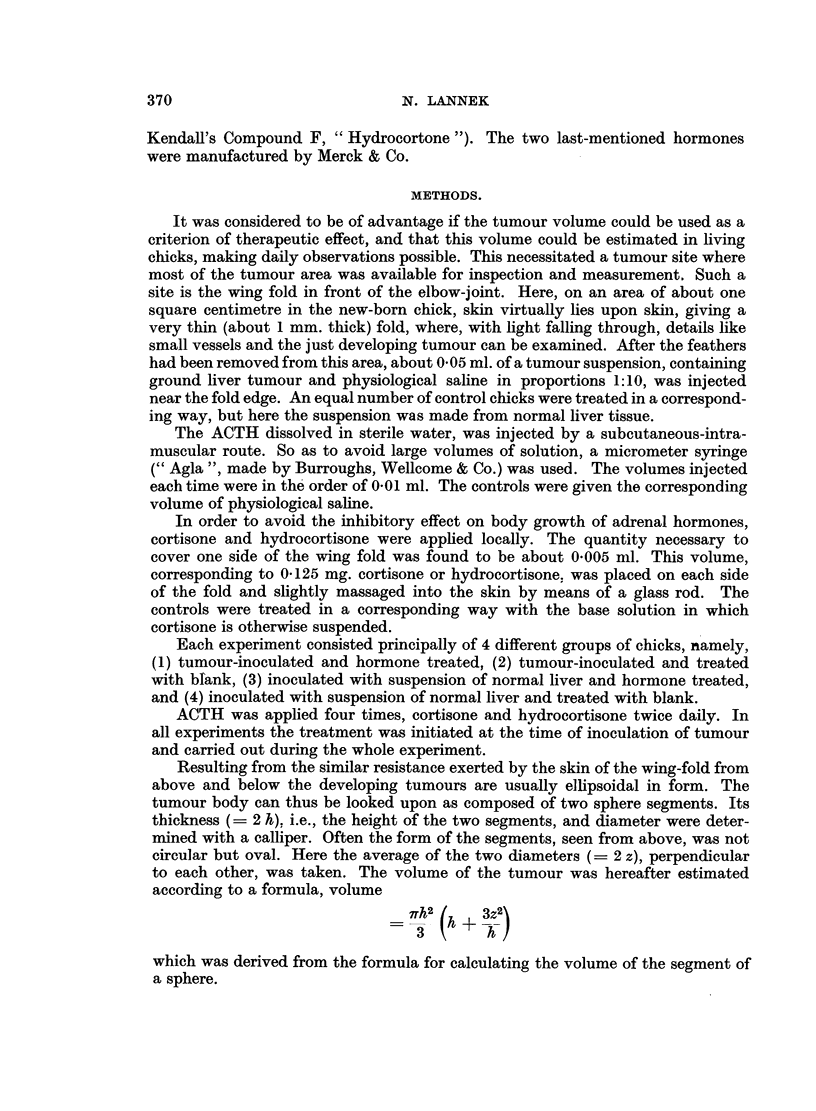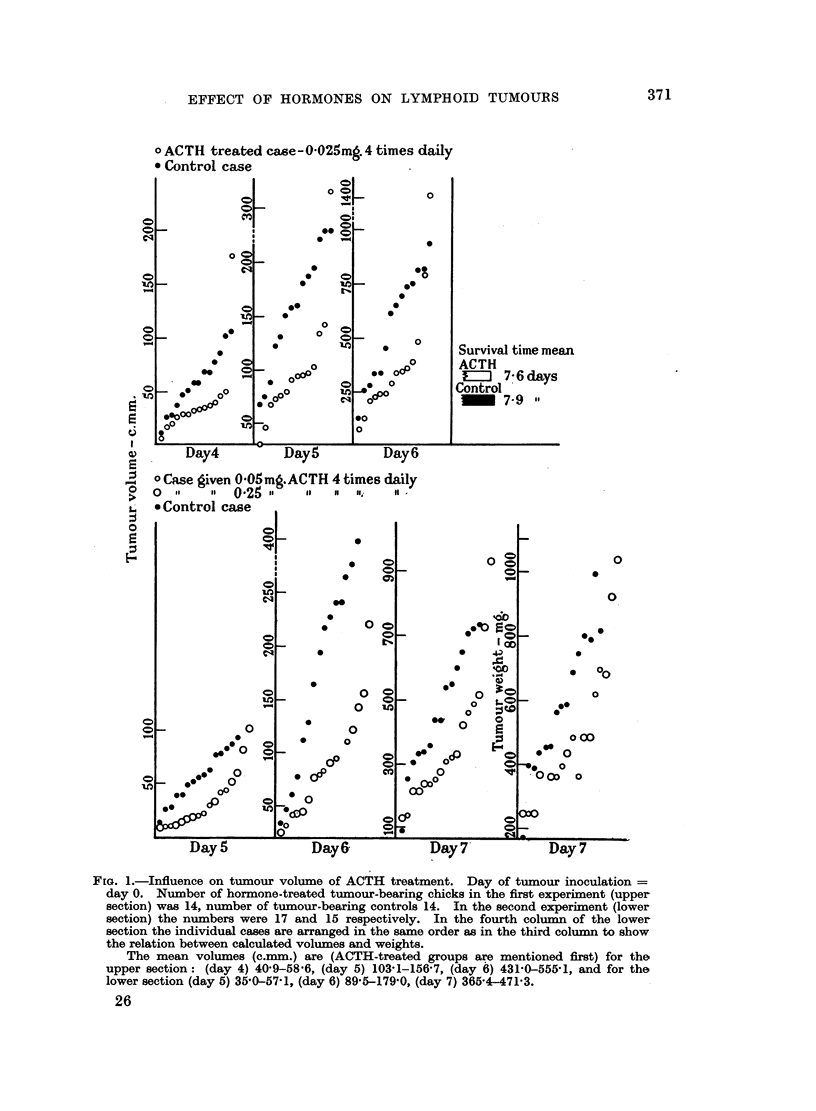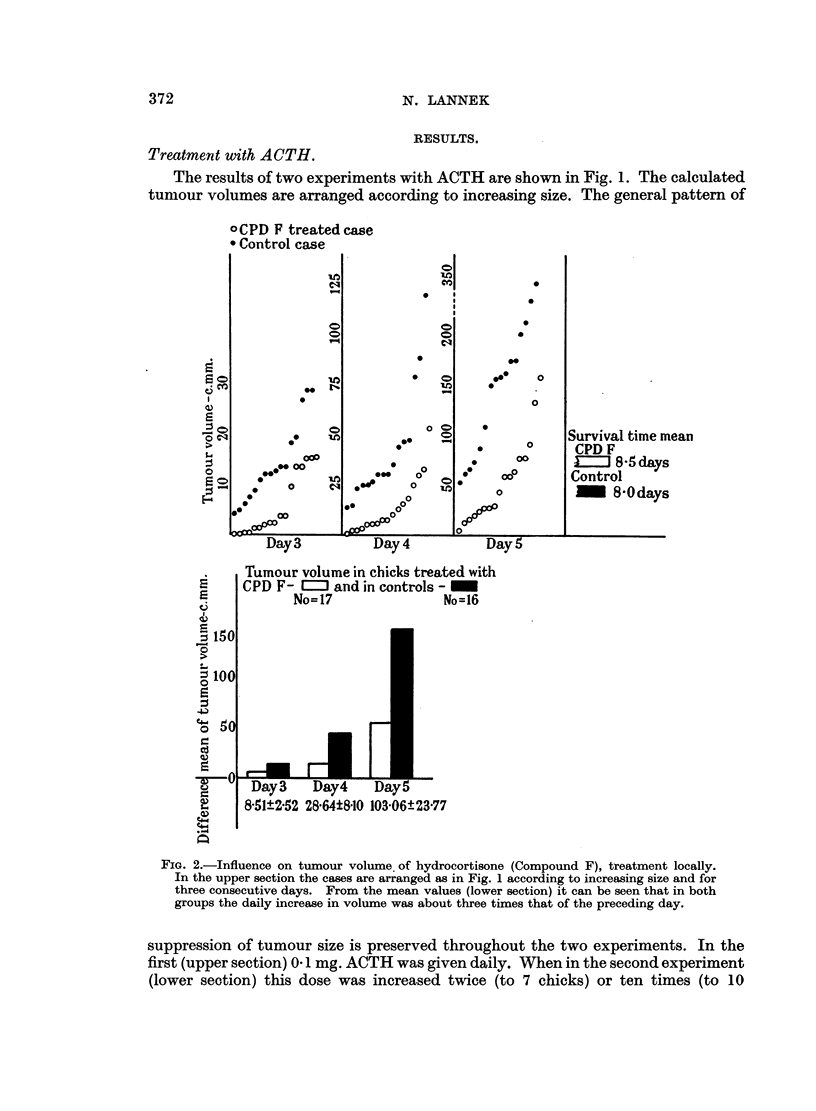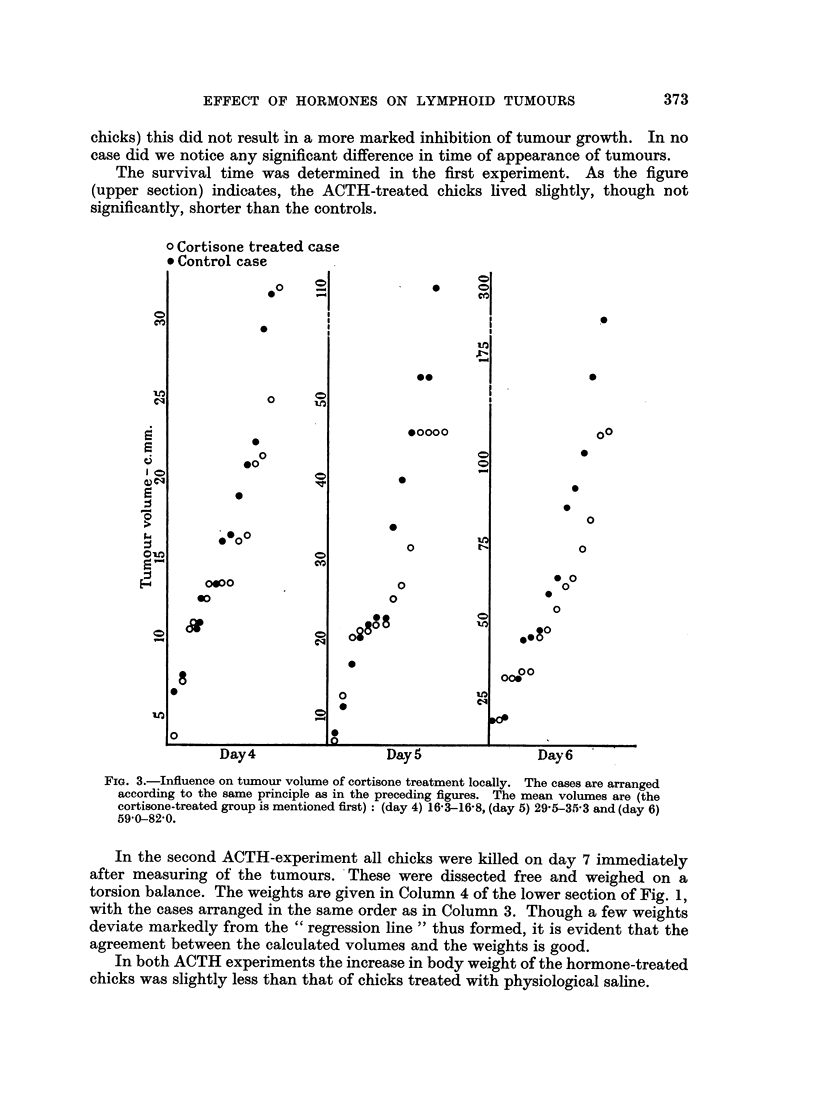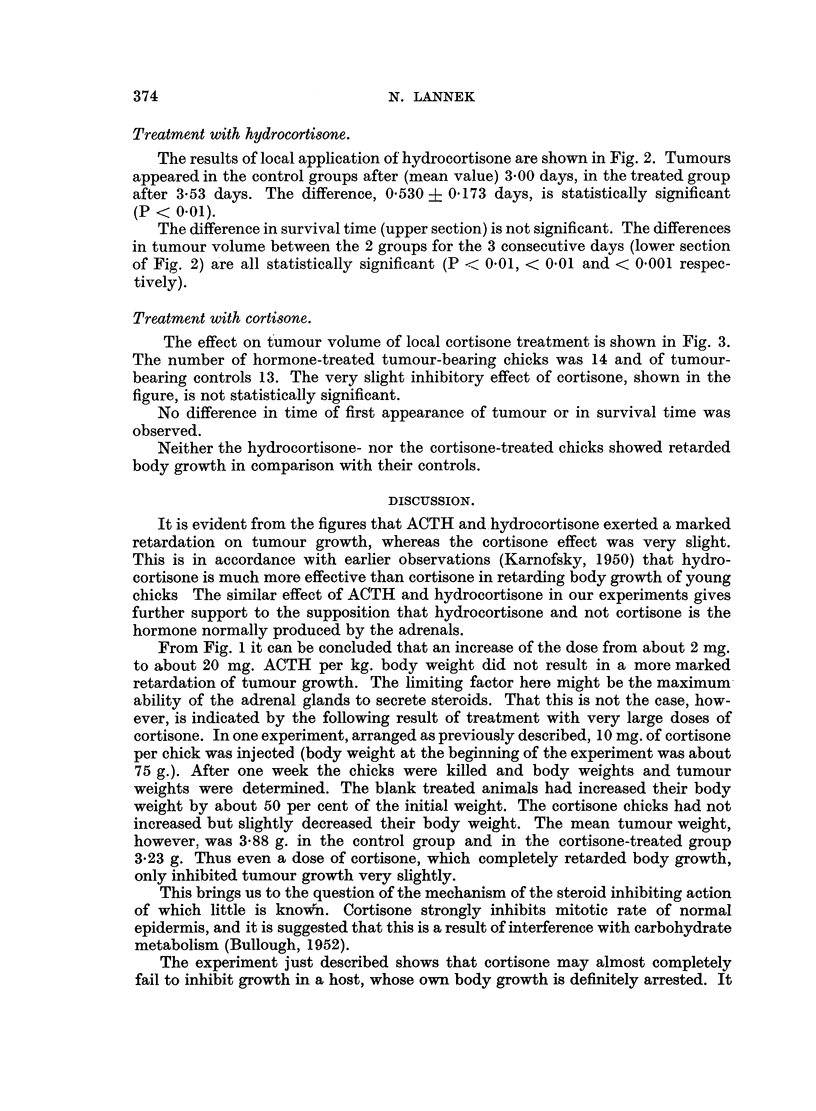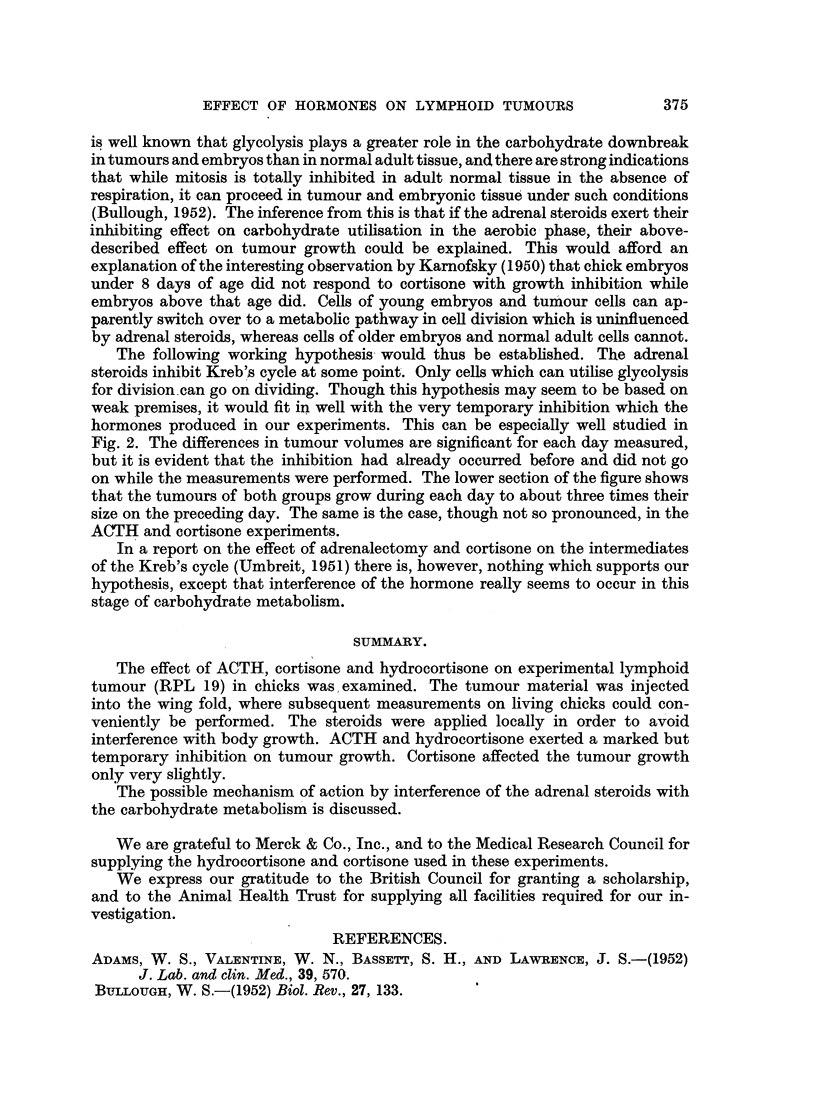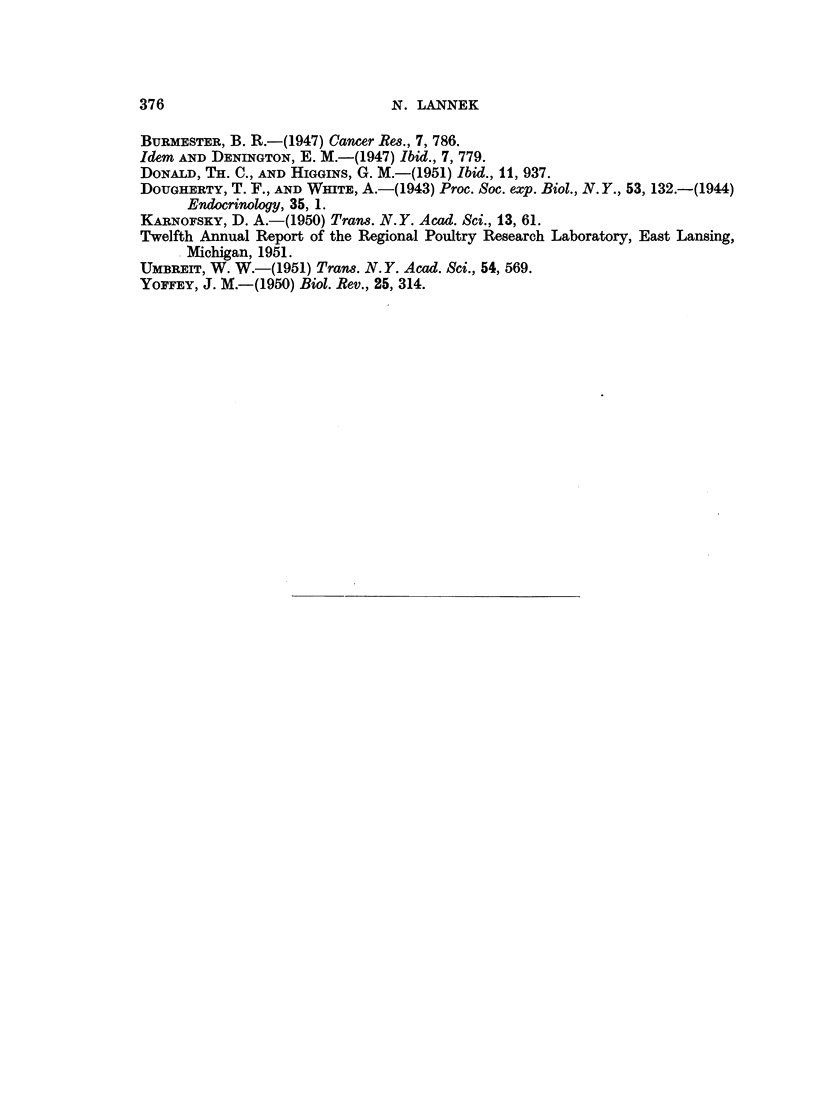# The Effect of Adrenocorticotrophic Hormone (ACTH), Cortisone and Hydro-Cortisone on the Growth of Experimental Lymphoid Tumours in Chicks

**DOI:** 10.1038/bjc.1952.41

**Published:** 1952-12

**Authors:** N. Lannek


### THE EFFECT OF ADRENOCORTICOTROPHIC HORMONE (ACTH), CORTISONE AND HYDRO-CORTISONE ON THE GROWTH OF EXPERIMENTAL LYMPHOID TUMOURS IN CHICKS.

N. LANNEK.

*From the Poultry Research Station, Animal Health Trust, Houghton Grange, Huntingdon.*

Received for publication August 25, 1952.

A LIVELY interest in the relationship existing between lymphocytic leukaemia and lymphoid tumours on the one hand and the adrenal glands on the other, followed the observation that involution of normal lymphoid tissue and lymphopenia can be achieved by the administration of adrenocorticotrophic hormone (Dougherty and White, 1943) and adrenal steroids (Dougherty and White, 1944). A considerable mass of literature on the subject has accumulated. This has been reviewed elsewhere with regard to experimental lymphoid tumour (Donald and Higgins, 1951), human leukaemia (Adams, Valentine, Barratt and Lawrence, 1952) and normal lymphoid tissue (Yoffey, 1950). In humans the most encouraging effects of treatment with ACTH and adrenal steroids seem to have been temporary remissions, while some cases proved completely refractory. In experimental mammals the results likewise have varied between marked retardation of tumour growth during at least some period of treatment, decrease of tumour incidence after experimental transfer, and somewhat increased survival time, and, on the other hand, no notable change according to these criteria.

In chicks the effect of cortisone on the experimental lymphoid tumour Strain RPL 12 was investigated (Twelfth Annual Report of the Regional Poultry Research Laboratory, 1951). A dose of 5 mg. was given daily in one group from the time of tumour appearance, and in another group beginning at tumour inoculation. No retarding effect on tumour-growth was observed but the treated birds died earlier than the controls, apparently as a result of bacterial infection.

In this report the influence of ACTH, cortisone and hydrocortisone, on the growth of the lymphoid tumour Strain RPL 19, will be described. This strain was developed from a case of spontaneous visceral lymphoid tumour (Burmester and Denington, 1947 ; Burmester, 1947).

### MATERIAL.

Crossbred Brown Leghorn × Plymouth Rock chicks, of both sexes, of 2 to 3 days of age were used. The sexes were randomised. The body weight of the chicks at the beginning of the experiments was approximately 40 g.

The hormone preparations used were adrenocorticotrophic hormone (ACTH RMC) manufactured by Roskilde Medical Company, cortisone acetate (also known as 11-dehydro-17-hydroxycorticosterone, Kendall's Compound E, " Cortone ") and hydrocortisone acetate (known alternatively as 17-hydroxycorticosterone,

Kendall's Compound F, " Hydrocortone "). The two last-mentioned hormones were manufactured by Merck & Co.

### METHODS.

It was considered to be of advantage if the tumour volume could be used as a criterion of therapeutic effect, and that this volume could be estimated in living chicks, making daily observations possible. This necessitated a tumour site where most of the tumour area was available for inspection and measurement. Such a site is the wing fold in front of the elbow-joint. Here, on an area of about one square centimetre in the new-born chick, skin virtually lies upon skin, giving a very thin (about 1 mm. thick) fold, where, with light falling through, details like small vessels and the just developing tumour can be examined. After the feathers had been removed from this area, about 0·05 ml. of a tumour suspension, containing ground liver tumour and physiological saline in proportions 1:10, was injected near the fold edge. An equal number of control chicks were treated in a corresponding way, but here the suspension was made from normal liver tissue.

The ACTH dissolved in sterile water, was injected by a subcutaneous-intramuscular route. So as to avoid large volumes of solution, a micrometer syringe (" Agla ", made by Burroughs, Wellcome & Co.) was used. The volumes injected each time were in the order of 0·01 ml. The controls were given the corresponding volume of physiological saline.

In order to avoid the inhibitory effect on body growth of adrenal hormones, cortisone and hydrocortisone were applied locally. The quantity necessary to cover one side of the wing fold was found to be about 0·005 ml. This volume, corresponding to 0·125 mg. cortisone or hydrocortisone, was placed on each side of the fold and slightly massaged into the skin by means of a glass rod. The controls were treated in a corresponding way with the base solution in which cortisone is otherwise suspended.

Each experiment consisted principally of 4 different groups of chicks, namely, (1) tumour-inoculated and hormone treated, (2) tumour-inoculated and treated with blank, (3) inoculated with suspension of normal liver and hormone treated, and (4) inoculated with suspension of normal liver and treated with blank.

ACTH was applied four times, cortisone and hydrocortisone twice daily. In all experiments the treatment was initiated at the time of inoculation of tumour and carried out during the whole experiment.

Resulting from the similar resistance exerted by the skin of the wing-fold from above and below the developing tumours are usually ellipsoidal in form. The tumour body can thus be looked upon as composed of two sphere segments. Its thickness ($= 2\,h$), i.e., the height of the two segments, and diameter were determined with a calliper. Often the form of the segments, seen from above, was not circular but oval. Here the average of the two diameters ($= 2\,z$), perpendicular to each other, was taken. The volume of the tumour was hereafter estimated according to a formula, volume

$$= \frac{\pi h^2}{3} \left(h + \frac{3z^2}{h}\right)$$

which was derived from the formula for calculating the volume of the segment of a sphere.

FIG. 1.—Influence on tumour volume of ACTH treatment. Day of tumour inoculation = day 0. Number of hormone-treated tumour-bearing chicks in the first experiment (upper section) was 14, number of tumour-bearing controls 14. In the second experiment (lower section) the numbers were 17 and 15 respectively. In the fourth column of the lower section the individual cases are arranged in the same order as in the third column to show the relation between calculated volumes and weights.

The mean volumes (c.mm.) are (ACTH-treated groups are mentioned first) for the upper section: (day 4) 40·9–58·6, (day 5) 103·1–156·7, (day 6) 431·0–555·1, and for the lower section (day 5) 35·0–57·1, (day 6) 89·5–179·0, (day 7) 365·4–471·3.

### RESULTS.

*Treatment with ACTH.*

The results of two experiments with ACTH are shown in Fig. 1. The calculated tumour volumes are arranged according to increasing size. The general pattern of

FIG. 2.—Influence on tumour volume of hydrocortisone (Compound F), treatment locally. In the upper section the cases are arranged as in Fig. 1 according to increasing size and for three consecutive days. From the mean values (lower section) it can be seen that in both groups the daily increase in volume was about three times that of the preceding day.

suppression of tumour size is preserved throughout the two experiments. In the first (upper section) 0·1 mg. ACTH was given daily. When in the second experiment (lower section) this dose was increased twice (to 7 chicks) or ten times (to 10

chicks) this did not result in a more marked inhibition of tumour growth. In no case did we notice any significant difference in time of appearance of tumours.

The survival time was determined in the first experiment. As the figure (upper section) indicates, the ACTH-treated chicks lived slightly, though not significantly, shorter than the controls.

FIG. 3.—Influence on tumour volume of cortisone treatment locally. The cases are arranged according to the same principle as in the preceding figures. The mean volumes are (the cortisone-treated group is mentioned first) : (day 4) 16·3–16·8, (day 5) 29·5–35·3 and (day 6) 59·0–82·0.

In the second ACTH-experiment all chicks were killed on day 7 immediately after measuring of the tumours. These were dissected free and weighed on a torsion balance. The weights are given in Column 4 of the lower section of Fig. 1, with the cases arranged in the same order as in Column 3. Though a few weights deviate markedly from the " regression line " thus formed, it is evident that the agreement between the calculated volumes and the weights is good.

In both ACTH experiments the increase in body weight of the hormone-treated chicks was slightly less than that of chicks treated with physiological saline.

*Treatment with hydrocortisone.*

The results of local application of hydrocortisone are shown in Fig. 2. Tumours appeared in the control groups after (mean value) 3·00 days, in the treated group after 3·53 days. The difference, 0·530 ± 0·173 days, is statistically significant ($P < 0{\cdot}01$).

The difference in survival time (upper section) is not significant. The differences in tumour volume between the 2 groups for the 3 consecutive days (lower section of Fig. 2) are all statistically significant ($P < 0{\cdot}01$, $< 0{\cdot}01$ and $< 0{\cdot}001$ respectively).

*Treatment with cortisone.*

The effect on tumour volume of local cortisone treatment is shown in Fig. 3. The number of hormone-treated tumour-bearing chicks was 14 and of tumour-bearing controls 13. The very slight inhibitory effect of cortisone, shown in the figure, is not statistically significant.

No difference in time of first appearance of tumour or in survival time was observed.

Neither the hydrocortisone- nor the cortisone-treated chicks showed retarded body growth in comparison with their controls.

### DISCUSSION.

It is evident from the figures that ACTH and hydrocortisone exerted a marked retardation on tumour growth, whereas the cortisone effect was very slight. This is in accordance with earlier observations (Karnofsky, 1950) that hydrocortisone is much more effective than cortisone in retarding body growth of young chicks The similar effect of ACTH and hydrocortisone in our experiments gives further support to the supposition that hydrocortisone and not cortisone is the hormone normally produced by the adrenals.

From Fig. 1 it can be concluded that an increase of the dose from about 2 mg. to about 20 mg. ACTH per kg. body weight did not result in a more marked retardation of tumour growth. The limiting factor here might be the maximum ability of the adrenal glands to secrete steroids. That this is not the case, however, is indicated by the following result of treatment with very large doses of cortisone. In one experiment, arranged as previously described, 10 mg. of cortisone per chick was injected (body weight at the beginning of the experiment was about 75 g.). After one week the chicks were killed and body weights and tumour weights were determined. The blank treated animals had increased their body weight by about 50 per cent of the initial weight. The cortisone chicks had not increased but slightly decreased their body weight. The mean tumour weight, however, was 3·88 g. in the control group and in the cortisone-treated group 3·23 g. Thus even a dose of cortisone, which completely retarded body growth, only inhibited tumour growth very slightly.

This brings us to the question of the mechanism of the steroid inhibiting action of which little is known. Cortisone strongly inhibits mitotic rate of normal epidermis, and it is suggested that this is a result of interference with carbohydrate metabolism (Bullough, 1952).

The experiment just described shows that cortisone may almost completely fail to inhibit growth in a host, whose own body growth is definitely arrested. It is well known that glycolysis plays a greater role in the carbohydrate downbreak in tumours and embryos than in normal adult tissue, and there are strong indications that while mitosis is totally inhibited in adult normal tissue in the absence of respiration, it can proceed in tumour and embryonic tissue under such conditions (Bullough, 1952). The inference from this is that if the adrenal steroids exert their inhibiting effect on carbohydrate utilisation in the aerobic phase, their above-described effect on tumour growth could be explained. This would afford an explanation of the interesting observation by Karnofsky (1950) that chick embryos under 8 days of age did not respond to cortisone with growth inhibition while embryos above that age did. Cells of young embryos and tumour cells can apparently switch over to a metabolic pathway in cell division which is uninfluenced by adrenal steroids, whereas cells of older embryos and normal adult cells cannot.

The following working hypothesis would thus be established. The adrenal steroids inhibit Kreb's cycle at some point. Only cells which can utilise glycolysis for division can go on dividing. Though this hypothesis may seem to be based on weak premises, it would fit in well with the very temporary inhibition which the hormones produced in our experiments. This can be especially well studied in Fig. 2. The differences in tumour volumes are significant for each day measured, but it is evident that the inhibition had already occurred before and did not go on while the measurements were performed. The lower section of the figure shows that the tumours of both groups grow during each day to about three times their size on the preceding day. The same is the case, though not so pronounced, in the ACTH and cortisone experiments.

In a report on the effect of adrenalectomy and cortisone on the intermediates of the Kreb's cycle (Umbreit, 1951) there is, however, nothing which supports our hypothesis, except that interference of the hormone really seems to occur in this stage of carbohydrate metabolism.

### SUMMARY.

The effect of ACTH, cortisone and hydrocortisone on experimental lymphoid tumour (RPL 19) in chicks was examined. The tumour material was injected into the wing fold, where subsequent measurements on living chicks could conveniently be performed. The steroids were applied locally in order to avoid interference with body growth. ACTH and hydrocortisone exerted a marked but temporary inhibition on tumour growth. Cortisone affected the tumour growth only very slightly.

The possible mechanism of action by interference of the adrenal steroids with the carbohydrate metabolism is discussed.

We are grateful to Merck & Co., Inc., and to the Medical Research Council for supplying the hydrocortisone and cortisone used in these experiments.

We express our gratitude to the British Council for granting a scholarship, and to the Animal Health Trust for supplying all facilities required for our investigation.

### REFERENCES.

Adams, W. S., Valentine, W. N., Bassett, S. H., and Lawrence, J. S.—(1952) *J. Lab. and clin. Med.*, **39**, 570.

Bullough, W. S.—(1952) *Biol. Rev.*, **27**, 133.

BURMESTER, B. R.—(1947) *Cancer Res.*, **7**, 786.
*Idem* AND DENINGTON, E. M.—(1947) *Ibid.*, **7**, 779.
DONALD, TH. C., AND HIGGINS, G. M.—(1951) *Ibid.*, **11**, 937.
DOUGHERTY, T. F., AND WHITE, A.—(1943) *Proc. Soc. exp. Biol., N.Y.*, **53**, 132.—(1944) *Endocrinology*, **35**, 1.
KARNOFSKY, D. A.—(1950) *Trans. N.Y. Acad. Sci.*, **13**, 61.
Twelfth Annual Report of the Regional Poultry Research Laboratory, East Lansing, Michigan, 1951.
UMBREIT, W. W.—(1951) *Trans. N.Y. Acad. Sci.*, **54**, 569.
YOFFEY, J. M.—(1950) *Biol. Rev.*, **25**, 314.